# Health, wealth and behavioural change: an exploration of role responsibilities in the wake of epigenetics

**DOI:** 10.1007/s12687-017-0315-7

**Published:** 2017-07-18

**Authors:** Danya F. Vears, Flavio D’Abramo

**Affiliations:** 1 Center for Biomedical Ethics and Law, Department of Public Health and Primary Care, KU Leuven, Leuven, Belgium; 2 Leuven Institute for Human Genomics and Society, Leuven, Belgium; 3 Freie Universität Berlin, Focus Area DynAge, Berlin, Germany

**Keywords:** Epigenomics, Social values, Informed consent, Social discrimination, Research subjects, Research ethics

## Abstract

The field of epigenetics is leading to new conceptualizations of the role of environmental factors in health and genetic disease. Although more evidence is required, epigenetic mechanisms are being implicated in the link between low socioeconomic status and poor health status. Epigenetic phenomena work in a number of ways: they can be established early in development, transmitted from previous generations and/or responsive to environmental factors. Knowledge about these types of epigenetic traits might therefore allow us to move away from a genetic deterministic perspective, and provide individuals with the opportunity to change their health status. Although this could be equated with patient empowerment, it could also lead to stigmatization and discrimination where individuals are deemed responsible for their health, even if they are not in social situations where they are able to enact change that would alter their health status. In this paper, we will explore the responsibilities of different actors in the healthcare sphere in relation to epigenetics across four different contexts: (1) genetic research, (2) clinical practice, (3) prenatal care and (4) the workplace. Within this exploration of role responsibilities, we will also discuss the potential constraints that might prevent the patient, mother-to-be, research participant or employee, from enacting any necessary steps in order to increase their health status in response to epigenetic information.

## Introduction

Epigenetics encompasses interactions between living conditions, lifestyle, gene expression and health whose effects might be inherited by future generations (Gilbert and Epel 2009). The field of epigenetics has become consolidated over the last decade for several reasons. The first driving factor was the ‘failure’ of the Human Genome Project to assist us in a complete understanding of the nature of all genetic disease. This led to the conceptualization that genetics alone cannot explain the most basic dynamics of living beings, such as how inheritance works (Maher 2008). In addition, technological advancements brought about within ‘omics’ disciplines has led to the transfer of their methods and approaches into biological laboratories in order to realize economies of scale (Hilgartner 2004; Rose and Rose 2013).

Despite its recent establishment as a field, in its early form, epigenetics developed about a century ago through the embryological studies carried out by Charles Manning Child, Conrad H. Waddington and Joseph Needham (D’Abramo 2017). The idea behind epigenetics consisted of considering organisms as a product of the interaction between genetic and environmental factors. Child, Waddington and Needham were politically engaged—Child was a biologist with reformatory ideas, whereas Waddington and Needham embraced the Marxist ideology to different degrees. This resulted in all three men placing a central emphasis on the environment, in both its material and social components. The principles behind epigenetics are that (i) environmental and behavioural factors, in a more or less direct manner, act at the biological/physiological level, such that these external factors elicit epigenetic dynamics that control and coordinate genes during all the developmental phases of the organism, and (ii) these biological dynamics controlled by behavioural and environmental factors are heritable to future cells and generations (Jablonka and Raz 2009). This is reminiscent of the historical Lamarckian concepts of inheritance of acquired characters, for which the initial proposal of epigenetics was, and still is, framed within the critical debate that considers the relationship between science and ideology (Gissis and Jablonka 2011; Jablonka and Lamb 2005).

As shown by Schicktanz, during the last decades, the social and political framework has changed deeply so that we can define the concept of *responsibility* in three phases (Schicktanz 2016). The first phase, from the 1960s on, was focused on *collective* responsibilities towards future generations, human kind or nature. In the second phase that started in the mid-1970s, responsibility was focused on *professional* responsibilities towards individuals, as shown by the rise of informed consent. In the third, starting in the 1990s, it was an intertwining of *social and individual responsibility*, a trend that mirrored a reaction to political reforms cutting back public welfare and health care (Schicktanz 2016).

From a technical perspective, the term ‘epigenetics’ refers to mechanisms involved in the regulation of cell type, transcription within specific tissues or expression of genes where there is no change in the DNA sequence (Ku et al. 2011). There are a number of ways in which this non-DNA gene regulation can occur. One biochemical modification involved is DNA methylation, where a methyl group is added to part of the DNA sequence, leading to activation or repression of the transcription of that gene (Ku et al. 2011). Regulation can also occur through histone modification, nucleosome positioning and expression of non-coding RNAs, among other mechanisms. The result of these changes is the winding, unwinding and clumping of the DNA which alter the degrees to which certain genes are expressed (Ku et al. 2011).

There are several ways in which these epigenetic changes are thought to relate to the development of disease. One predominant theory relates to the developmental origins of health and disease (DOHaD) hypothesis in which exposure to external factors during critical developmental phases, often in utero, are thought to influence an organism’s predisposition to disease (Barker 2007). These environmental factors can take a number of forms, such as the presence of pathogens, exposure to toxins and the availability of nutrients and water (Bateson et al. 2004). The theory is that during a ‘critical period when a system is plastic and sensitive to the environment’, this exposure takes place which ‘programs’ the genome of the organism to function at a certain capacity through these epigenetic regulatory components (Barker 2007). Consider, for example, a pregnant woman who is undernourished. The exposure of the fetus to the reduced levels of nutrition it is receiving from the mother is thought to lead to changes in the metabolic interaction between the mother and the fetus in different ways, depending on (a) when it happens during fetal development and (b) how prolonged the period of undernourishment lasts (Barker et al. 1993). These metabolic changes relate to growth hormones which can affect the development of a number of different tissues, such as the development of the pancreatic cells and also the vascular system, as well as affect placental and/or fetal growth which results in a smaller baby at term (Barker et al. 1993). While in some cases these changes might be transient, often these critical periods of developmental plasticity are ‘followed by loss of plasticity and a fixed functional capacity’ (Barker 2007).

This exposure might then impact on the disease status of the fetus in a number of different ways. Barker uses his own research to highlight that programming that takes place during maternal undernourishment during critical periods of plasticity in pregnancy might lead to the poor development of the vascular system. This is not adaptive to the *future* environment of the fetus but may reflect how the fetus is developing in order to adapt to the reduced available nutrition (Barker 2007). The result is that the undernourished fetus may develop cardiovascular disease in adulthood, regardless of the environmental factors that it is exposed to after birth (Barker et al. 1993).

In contrast, there are other situations where, in conjunction with this early programming, the lifestyle or exposures of the adult may also contribute to the development of disease. An example of this would be the high incidence of non-insulin-dependent diabetes in people who had low weight at birth or during infancy but who developed obesity in adulthood (Barker et al. 1993). In this example, the programming was present which resulted from exposure to maternal undernourishment and subsequent changes in glucose-insulin metabolism during fetal development. Yet, the development of obesity and the challenge this presents to the pancreas lead to the onset of diabetes. This model of disease development has been labelled the ‘mismatch model’, because the rationale behind it is that the early programming in environments where food is in short supply might actually have an adaptive quality. However, when the environment changes, such as when food is in abundance, the programming becomes maladaptive and leads to the development of disease (Bateson et al. 2004).

At this stage, research investigating the potential for interventions in order to change our epigenomes to improve health status is still in its infancy and much of the evidence to date, particularly in relation to the potential for transgenerational inheritance of epigenetic phenomena, has come from animal studies (Joly et al. 2016). In addition, researchers working in the field hold quite divergent views about the significance of epigenetics, with some ‘champions’ believing that it is the key to understanding what we know from traditional genetics, and other, more skeptical researchers disagreeing that epigenetics drastically changes our knowledge in the field (Tolwinski 2013). Despite these reservations, the new-found knowledge of how epigenetics can impact on disease could have great power and there are hopes that it may provide us with an opportunity to move away from a genetic deterministic perspective and allow individuals the ability to change their health status (Canning 2008; Van de Vijver et al. 2002). While this could be equated with patient empowerment, we need to be aware that it could also lead to stigmatization and discrimination where individuals are deemed responsible for their health, even if they are not in social situations where they are able to enact changes that could alter their health status. Given that epigenetics is already receiving considerable media coverage (Lappe 2016), the concerns about potential misunderstandings, discrimination and stigmatization need careful consideration sooner rather than later (Cozzens and Woodhouse 1995).

In addition, we need to be aware of the potential for the field of epigenetics to get stuck in adopting a ‘technical fix’ approach. This trend, which has developed over the last decades due to the collaboration between the private/financial sector and the public institutions of research (Young et al. 2008), has changed the functioning and aims of research, leading to overlap between financial and academic aims (Cozzens and Woodhouse 1995; Etzkowitz and Webster 1995). Technological fixes are indeed instrumental to financial dynamics focused on handling societal problems within private corporate structures. This approach in turn fuels a deficit model where people are conceived as ignorant and in need of education regarding scientific arguments (Irwin and Wynne 2003; Wynne 2014). The concern is that epigenetics might also follow this trend where innovation (e.g. production of therapeutics and diagnostics through use of patents and intellectual property rights) might supersede public goods (e.g. policies to incentivize health promotion), which in turn could easily lead to a range of moral discourses subjecting women, patients and citizens to increased scrutiny (Kenney and Müller 2016; Meloni 2016a; Pickersgill 2016). In addition, the public may not want to be ‘educated’ in this regard and may react negatively to experts wanting to discipline them without the presence of shared values, which can hinder the fair translation of responsibilities in the public sphere.

The interaction of knowledge built by experts and reception of that knowledge by the public has been scrutinized in different manners, so that some categories/criteria were formulated to describe the more or less basic steps to understand allocation of responsibilities (Hedlund 2012; Schicktanz 2016). In order for an agent to be responsible for an action or situation, a number of criteria must be met. First, there needs to be a causal link between the agent and the situation under consideration (Young 2006). Second, they have to be aware, or cognizant, that their action caused the event (Hedlund 2012). Third, there needs to be a motivation for the agent to act in a certain way that is societally or culturally agreed upon (e.g. obligations, rewards, incentives, encouragement, etc.), rather than just based on the agent’s own will (Gilbert 1993; Hedlund 2012). And fourth, the agent needs to be able to exercise some degree of control over the situation and to be able to exercise autonomy in her choice to act (or not) to cause that action (Fischer 2006; Hedlund 2012). In relation to responsibilities in epigenetics, Dupras has warned against assigning epigenetic responsibilities too readily to individuals without proper consideration of ‘the ambiguous nature of epigenetic mechanisms’ (Dupras and Ravitsky 2016). Moreover, Schicktanz has highlighted genetic responsibility, that here we place on the same level as epigenetic responsibility, as a notion to identify the internalization of individual feelings of guilt or self-restriction (Schicktanz 2016). Likewise, in contrast with the normative position of Hedlund, Pickersgill and colleagues have argued, that biomedical research in epigenetics will create further ways in which individuals can be made responsible, as caretakers of life that does not yet exist (Pickersgill 2016). We will not attempt to provide any specific solution to the issues raised in this paper, as we think political problems need to be addressed by local communities in order to initiate a negotiation with both public and private scientific institutions. A common idea runs through all four contexts analysed below that relates to the social, political, behavioural and environmental factors as determinants of health. This idea that the context influences, determines or causes biological and health changes traces back to Hippocrates, among others, who more than two thousand years ago described environmental, social and political factors as determinants of health (Jones 1957). Jean-Baptiste de Lamarck and, to a lesser extent, Charles Darwin also focused on the effect of environmental conditions on biological variations. More recently, institutions like the World Health Organization (WHO) and the International Agency for Research on Cancer (IARC) have developed analyses and interventions around the relevance of social factors on health (i.e. working conditions, diet, educations, poverty, living habits, education, etc.) (James and Ronald 2012; Marmot 2015; Marmot and Wilkinson 2005; Tomatis 1997; World Health Organization 2013). In addition, challenging programs on epigenetics and DOHaD are pointing precisely at the manner in which social and material context modulates health of humans (Párrizas et al. 2012; Rosenfeld 2015), for instance, how globalization might impact on epigenetic patterns and non-communicable diseases (Vineis et al. 2014). With this in mind, in this paper, we explore the responsibilities of different actors in the healthcare sphere in relation to epigenetic testing across four different contexts: (1) genetic research, (2) clinical diagnostics, (3) prenatal care and 4) the workplace; and discuss the potential constraints that might prevent the patient, research participant, employee or mother-to-be, from enacting any necessary steps in order to increase their health status based on epigenetic information.

## Scenario 1—genetic research

A research team is conducting a study investigating the impact of night shifts on risk of developing breast cancer. The team explores the hypothesis that the disruption of the circadian rhythms caused by working at night, and the exposure to the lighting used in these workplaces, alters patterns of gene expression and melatonin homeostasis, leading to the development of cancer. This is based on previous research showing an association between night shifts, circadian rhythms and breast cancer (Fenga 2016; IARC 2010a; Reszka and Przybek 2016; Stevens 2009; Straif et al. 2014). This association may be explained either as deregulation of the genes’ expression for the changes of endocrine levels caused by working at nights or as the effect of the presence of some genetic polymorphisms in the circadian pathway genes responsible for increasing breast cancer risk when triggered by disruption of circadian rhythms.

The project, as with many other scientific endeavours, is a public-private partnership (Meslin et al. 2015; Perkmann et al. 2013). In order to conduct it, the research team sets up a biobank of biological samples from shift workers which will comprise blood and hair. The DNA from the samples will be analysed to look for single nucleotide polymorphisms (SNPs) and epigenetic patterns of the genes’ expression using genomic sequencing (GS). The findings of the research may lead to new insights into policy-making for cancer prevention or potential innovative treatments for cancer patients.

One unclear aspect of this research relates to the proximity of the two hypotheses of the project. While apparently complementary, they address the problem in two different manners. An epigenetic approach might focus on links between gene expression, endocrine factors, circadian rhythms and working at night. Within this model, the individuals’ physiology might be considered in order to develop interventions at either the environmental level, such as reducing shifts and altering the lighting of the working place, or at the endocrine level, such as producing pharmaceutical agents able to restore the levels of melatonin and estrogen which leads to a deregulation of genes expression underpinning cancer initiation and development. Instead, the genetic explanation based on genome-wide association studies (GWAS), conducted to discover single nucleotide polymorphisms associated with cancer susceptibility, confers to women a predisposition based on innate, genetic characteristics. This could lead researchers to discover genetic pathways to act upon through the use of targeted drugs. These two approaches support two different models, one which considers individuals as dynamic systems changing together with their environments, and another considering individuals as a fixed nexus of mechanisms, mainly determined by their genes or epigenetic characteristics.

But are the two approaches really complementary or are they opposed? The erroneous presupposition here consists of conferring certain powers to certain specific technologies and models of causation, so that epigenetic and epigenomic analyses using GS should lead to an epistemic, causal justice, where models and practices utilized by scientists grant ontological primacy to DNA—i.e. the phenotype is the result of *either* environment or genotype. It is instead necessary to consider a more dynamic and comprehensive relationship between individuals and their environments, in order to overcome causal impasses affecting the possibility to formulate an aetiological explanation, i.e. the phenotype is the result of the interaction between environment and genotype, and in no case are two genotypes identical in their reactions (Lewontin 2006; Waddington 1953). Both genotypes and environments are causes of phenotypic variations, and as such necessary objects of study to understand phenotypes or diseases (D’Abramo 2014). As both Richardson and Meloni highlight, the modern programs of research on human epigenetics do not challenge genetic determinism and biological reductionism. Instead, epigenetics might be used to pathologise the poor or reinforce the biological differences or inferiority of individuals living in disadvantaged social conditions (Meloni 2016a; Richardson 2015). When epigenetics considers either the genotype or the environment, it might easily lead to discrimination by allocating responsibilities to (biological functioning of) individuals without producing any increase in power to impact on social, individual or physiological determinants of health. Indeed, epigenetics may rely on empirical evidences produced within laboratories where the foreseen interventions are mainly conceived at the molecular level. How the new postgenomic science of epigenetics will allocate responsibilities to realize particular types of social justice, after having molecularised the social milieu and biographies of individuals (Niewöhner 2011), is yet to be determined (Del Savio et al. 2015; Loi et al. 2013; Waggoner and Uller 2015). Allocation of social and individual responsibilities through scientific research also pertains to perspectives of *longue durée*, where the metaphysical presupposition of translating social and cultural issues in molecular terms formulated some decades ago (Hacking 1995; Waddington 1967) will propel part of the future biomedical research.

In order to disentangle the social effects of scientific practices, it might be useful to consider the roles and interactions among responsible stakeholders. A matrix that heuristically inspired the analysis of the case here presented was recently sketched in respect of ‘genetic risk and responsibility’ (Schicktanz 2016). In our scenario, the main actor is a hypothetical principal investigator (PI). The PI is constrained, both through the working contract they sign when they commence their role and the evaluation processes of scientific research, of which dissemination of findings is a significant component. If the research project is carried out using public funds and infrastructures, it is fair to expect results to benefit taxpayers who indirectly fund biomedical research. Therefore, as a *moral agent*, the PI has a responsibility towards a *moral object*, the taxpayers, and is supervised by ethical committees, institutional review boards and bureaucratic mechanisms. The standards he applies are derived from scientific customs, or research ethos, and have certain *consequences* that are framed within a precarious labour market. The principal investigator also has the burden of securing both his own salary and the wages of the research team. However, determining how to balance the responsibilities of the PI towards taxpayers and the workers engaged in medical research is a complex issue. In fact, it is likely that some conflicts between these two social responsibilities might arise. Imagine that the PI secured private funds through a pharmaceutical company which he uses to pay the postdoctoral researchers. Also, imagine that he discourages researchers from scrutinizing results that suggest that epigenetic factors increasing the women’s risk of breast cancer could be reversed by altering the night shifts themselves and encourages them to focus on results that suggest a potential for pharmacological interventions. The PI wants to secure future funds from the same foundation and is therefore prone to please the interests of the foundation trustees. In other words, the principal investigator wants to give his ‘scientific’ contribution to support the working place’s profits through the intensive pace of production required. With the best of intentions, the principal investigator is primarily concerned about his own salary and of his team. In pleasing the funding body by excluding some hypotheses from the project, is he being unfair to research participants that are also taxpayers? And if so, is the principal investigator accountable for having subordinate subjects of research to job positions of his research team? Here, it seems that some aspects characteristic of funding bodies and of hierarchical order of biomedical research might narrow the possible gamut of hypotheses, and eventual solutions, for social medical problems, to legitimize a deterministic stance (i.e. that problems derived from social conditions like working at night are mainly biological problems) by means of anti-reductionistic, postgenomic approaches.

Another problem researchers might face is of epistemic nature and relates to the possibility of reversing epigenetic dynamics. The debate, on which there is no consensus, surrounds the possibility of reducing social dynamics to biological ones; diseases derived from certain working conditions are reduced to biomedical problems. Based on the answer to whether it is possible to reverse specific epigenetic biological factors in women who develop breast cancer because they work night shifts, and on the fact that ethics committees often do not encourage dissemination of findings apart from in scientific articles, researchers will decide if it is worth communicating the results of the study to the women engaged in the research. The question of reversibility of epigenetic factors is related to the aims of the research themselves. If researchers also consider the possibility of influencing those who are empowered to influence policy relating to the frequency and length of night shifts, then the possibility of addressing the problem might materialize. The potential to find a solution might then increase not only the desire but also the responsibility of researchers to communicate the findings of the research to participants. Nevertheless, the PI’s drive to secure future funding by pleasing the funding body might easily translate into a reluctance to consider other solutions which would alter the current high production rhythms of workers.

If researchers are not inhibited by this ‘pleasing chain’ of the precarious labour market (i.e. doing research to support policies dismantling public welfare and healthcare systems), they might instead aim to identify primary interventions from their research findings, in order to prevent women working night shifts from developing cancer. They might then consider communicating the outcomes of their research to employers and policy-makers to contribute to a negotiation between employers and employees. Both the genetic and epigenetic models we described, the former indicating that women who might develop cancer because of their genetic makeup, and the latter indicating the incidence showed by cohort studies engaging women working in specific working settings, could be used to develop primary interventions, and make policies addressing the safety of workers. What if, however, there is no other manner to address cancer predisposition aside from by not working at night? Is it responsible for researchers to communicate a risk for which no solutions are envisaged and that might eventually result in a deterioration of the participants’ social factors? For example, workers who live in an area where there is a high rate of unemployment might be faced with the choice to either work or be healthy.

In addition, researchers might be constrained in their ability to communicate the specific aims of the study to participants, because the research aims of investigating genetic polymorphisms and epigenetic patterns associated with cancer initiation and development may not have any direct translational outcomes for policy, diagnostics, or therapeutics, at least not in the short/medium term. Therefore, even if researchers would like to communicate more specific aims with participants, they may not be able to do so, as they cannot foresee the translational or social value of their research (researchers mostly work to publish articles that might increase their chance to secure a future job). This lack of information in turn inhibits the participants’ ability to make autonomous decisions about entering the research study, as well as their ability to be actively engaged. This lack of engagement may then inhibit the researchers’ ability to ask participants for more information, for instance, to enrich the study with updated individual phenotypic data. These aspects of the research that are deeply determined by the nature of private-public partnership (i.e. the ‘unknowability’ of aims, inability to actively engage participants, lack of communication between researchers and participants, overlapping of social and for-profit/innovative aims, etc.) constrain the manner in which researchers create the scientific facts that will eventually be used to assign responsibility at certain levels.

Engagement of the private sector in biomedical research and epidemiology is not a novelty and is necessary to different degrees (e.g. technological tools that are supplied by corporations). A question that might help to shape a constructive debate regard the roles and modalities of engagement of the private sector in public health. Indeed, rather than the private nature of the funds, what creates the problem is the private nature of some dynamics which shapes the research, such as the PI shaping the aims of the research by excluding public health interventions to please the foundation’s trustee and anti-welfare policies. This impasse is tightly bound to issues related to labour market policies. One could imagine that using a broad consent approach, in which the aims, benefits and risks of research are not necessarily discussed in detail, would mean that the origin of funding and the research aims would not be disclosed (D’Abramo 2015; D’Abramo et al. 2015; Hofmann 2009). Most of the time, broad consent translates into secrecy about the for-profit nature of the research, whereas open disclosure of funding and the aims of the research might clarify the boundary between private and public interests (Jasanoff 2002; Krimsky 2005; Krimsky and Nader 2004). In turn, this aspect influences the role of scientists in their interaction with the public, so that other questions might be better addressed, such as how biomedical research can encourage an open dialogue across scientists, citizens, patients and stakeholders. However, is epigenetics, that was developed as a discipline which captures the dynamic, dialectical interaction between organisms and their environments, instead proposing a narrower concept of environment which cuts off those factors the supporters of anti-welfare reforms want to remain undisputed?

Indeed, lines of biomedical research are principally shaped through devices that, besides being a fair approach to the privatization of science, can also produce profits—i.e. data production, data sharing, patents and intellectual properties (Sunder Rajan 2006; Sunder Rajan and Leonelli 2013). Does it mean that medical research driven by a corporate logic is not capable of producing facts that underpin preventive, welfare-supporting policies? And if these preventive policies are then produced, are these policies, at any point, confronted with the values of participants of research? Are any of the outcomes of research co-constructed by researchers and lay people? These questions lead us to consideration of the role of patients and healthy recipients of preventive, diagnostics and therapeutic practices.

## Scenario 2—clinical care

A 52-year-old male goes to a general practitioner, because he is experiencing difficulty urinating during the day and also increased frequency of urination during the night. His father developed prostate cancer, and he is concerned that his symptoms are similar to those his father experienced. The doctor performs a digital rectum examination, which suggests some inflammation, and takes a blood sample for a prostate-specific antigen test. The doctor has been reading about new biomarkers for prostate cancer and decides to send a sample from the patient for a test to investigate DNA methylation. While the PSA is within the normal range, indicating the patient does not have prostate cancer, the DNA methylation test identifies that the patient has a higher than average level of global hypomethylation. This can lead to genomic instability, focal hypermethylation of promoter regions in tumour suppressor genes and, subsequently, a high risk of cancer. This hypomethylation could be due to a number of factors, including the fact that he grew up in a poor area, but also his tendency to smoke 40 cigarettes a day, his poor diet and heavy drinking. The doctor advises the patient that in order to reduce his risk of developing prostate cancer, as well as other forms of cancer, he should take steps to improve his lifestyle such as quit smoking, eat better and cut back his alcohol consumption. The doctor suggests that this may reverse some of the effects and reduce the patient’s cancer risk.

On the surface, some might view this information as empowering for the patient because it gives them the opportunity to enact change in their diet and lifestyle to increase their health. However, if we consider this more deeply, we can see that placing the responsibility on the individual here is problematic. As discussed previously, according to Hedlund, in order for an actor to be responsible, there are a number of components that are necessary: causation, cognizance, obligation and capacity (Hedlund 2012). One could argue that by performing this test, the healthcare professional has established a link between the patient’s behaviours and their risk of cancer, thereby fulfilling the first criteria, causation. By receiving the test results, the patient has been made aware of their increased risks, fulfilling the second criteria, cognizance. In addition, as the patient is seeking medical investigations in order to prevent developing a medical condition which would place additional burden on the healthcare system, some might argue that he has an obligation to enact change which will prevent the development of this condition in order to benefit future generations.

However, by stating that these three criteria are fulfilled carries with it a number of assumptions. First, it assumes that enough is known about the interactions between behavioural factors, such as smoking, drinking and diet, and their effect on DNA methylation to guide medical recommendations. However, to date, there is little in the way of evidence, particularly in humans, that epigenetic patterns can be altered through medical, lifestyle and/or chemical interventions. The doctor has also made the assumption that the results of the DNA methylation study are due to the patient’s current lifestyle behaviours and that by changing these behaviours, the cause of the epigenetic signature will be removed and that this will, in turn, lead to amelioration of their health. But what if the patient grew up in a position of low socioeconomic status, had poor nutrition from a young age and lived in an area with high levels of pollution? This early exposure could also be the cause of his epigenetic results, rather from his current lifestyle. It also assumes that the patient has understood the results of the test and the connections that the doctor is drawing between his lifestyle and his risk of cancer. However, understanding genetic and epigenetic risks represents a huge challenge for both laypersons and experts.

Third, the ‘obligation’ criteria assumes that there is a collective agreement about what constitutes ‘good epigenetic health’ and that this is something that one can strive for (Hedlund 2012). However, as Dupras points out, this is far from straightforward (Dupras and Ravitsky 2016). For example, it is possible that the patient’s epigenetic pattern is actually due to his exposures during fetal development. The mismatch model of disease development proposes that the fetus is, through the mother, exposed to the kind of environment that it is likely to be born into and the resulting epigenetic pattern is imprinted in order to allow better adaptation once they are born and throughout their lifespan (Bateson et al. 2004). Using this logic, the patient’s epigenetic pattern is not abnormal in and of itself. Rather, it is mismatched to the environment in which the patient is currently living. This theory means that there is no such thing as a ‘normal’ epigenome that one can aim for in order to achieve good epigenetic health (Dupras and Ravitsky 2016). In addition, while one can imagine that quitting smoking, eating a better diet and consuming less alcohol would have a positive impact on his health, there is currently insufficient knowledge in this field to conclude that, even if our patient made radical lifestyle changes as per the doctor’s recommendations, there would be any significant change to his global DNA methylation levels and any increased risk of cancer associated with this. This lack of evidence for an ability to alter DNA methylation patterns therefore signifies that with our current level of knowledge, the capacity criterion cannot be fulfilled.

Let us assume, however, that these criteria have actually been met in that the patient is actually the cause of his increased risk, is aware of it, that there is some concept of good epigenetic health he can aim for and that there are interventions available to reliable alter his epigenetic pattern. In order to assign responsibility to him for his health, we would still require that he was actually capable of doing something to change it (Hedlund 2012). But what level of control does the patient actually have to change their health status? Whether the patient has the capacity to make these changes is questionable, because individuals are embedded in different collectives, such as families, friendship groups or work places, and within these collectives, their choices are constrained in various ways (Mol 2008). For this reason, rather than a choice being an individual decision, it becomes a decision which is either facilitated or not, by the collectives in which the individual is embedded (Mol 2008). Perhaps our patient has a stressful job, works very long hours and has no wife or children. He has little time to make friends, and therefore, his only stress release is to go out after work with his colleagues, who also drink and smoke heavily. With these colleagues as his only support network, implementing behavioural changes that go against the behaviours of the collective is very difficult and might result in a situation of isolation and deprivation. Societal factors also impact on one’s capacity to implement change. Consider that it might have taken our patient 6 months from when he first developed symptoms to visit the doctor. While this delay could have eventuated due to his long working hours, making it difficult to attend appointments during the work day, it may also be culturally based as men (for reasons relating to social constraints, such as job status or gender role) are less likely to seek medical advice when they are ill (Baker et al. 2014).

While we have established that it would be unjustified for the epigenetic responsibility within this scenario to rest (solely) with the individual patient, we need to think about the responsibilities of other actors. We can, for example, consider the role, and therefore the responsibilities, of the doctor in this scenario. The role of a doctor is to promote the wellbeing of their patient primarily through beneficence and non-maleficence (Beauchamp and Childress 2001). Superficially, it may seem that the doctor is fulfilling his responsibility to the patient by ordering the epigenetic testing in order to determine the patient’s risk of cancer and provide them with the opportunity to implement behavioural change. However, if we consider the patient’s lack of capacity to enact this change because of the collectives in which he is embedded, then perhaps the doctor is actually doing more harm than good by ordering epigenetic testing and disclosing the results to the patient. If the medical information is not *realistically* actionable, disclosure of the results from the epigenomic test could easily lead to an increase of the patient’s concerns and stress. If we also consider the nature of the lifestyle recommendations provided, one might question whether the doctor would have suggested anything differently, regardless of the test outcomes. One might also suggest that it is a component of the doctor’s role to take the situation of the individual patient into account by assessing how these ‘unhealthy behaviours’ might be created by their social situations and therefore how their ability to implement change might be constrained.

It is also important to consider what impact this knowledge is likely to have on the patient. They may feel empowered by the knowledge that they have an increased risk of developing cancer, because they have the potential to do something about it. But what if he does take steps to improve his health and a repeat methylation test shows no difference? This failure, despite his attempts at compliance, is not likely to empower him to take steps to improve his health in the future. What if the patient does not make lifestyle changes and he develops cancer? Is he more responsible for his health status than someone who has not had their epigenome tested, because they were informed about their risks? In order for an individual to bear more responsibility, they must also be given more power. Therefore, just giving the patient information is not enough to increase their level of responsibility. Despite this, although it may be unjustified to place the responsibility on an individual for their epigenetic health, such that they should be blamed for their ill health if it eventuates, it might still be beneficial to empower individuals to take better care of themselves generally as this could lead to disease prevention, seeking help which may result in early identification, and potentially access to a broader range of possible treatments.

In addition to caring for patient wellbeing, over time, there has also been a shift, driven by patient preferences, from paternalistic models of care to those which have a greater focus on patient autonomy and self-determination (McCoy 2008; Quill and Brody 1996). This shift to promote autonomy should entail providing the patient with the ability to give informed consent for the test. Given the complex nature of epigenetics, one can imagine that it would be difficult to explain the potential outcomes, including the potential for incidental findings, related to the test in sufficient detail for the patient to make an informed decision about submitting their sample for testing.

Of course, there are also financial implications associated with using this technology which need to be considered. On one hand, the information about the patient’s increased risk of cancer could be used to benefit the patient. If they did develop cancer, then perhaps they would be entitled to reduced rates for their investigations, treatments and general medical expenses because they were ‘epigenetically disadvantaged’. But would this still be justified if the patients were informed that they were at risk, had the knowledge of how to reduce their risk and chose not to change their behaviours? On the other hand, one could foresee health insurance companies using this kind of information to their advantage and charging higher premiums to those who were deemed to be more at risk based on their epigenetic profiles, similar to their current practices relating to asking consumers about their smoking behaviours and family history. If we consider what are the responsibilities of insurance companies in this situation, one could argue that they are responsible both for providing the service the consumer is paying for, and also for the way they charge for that service to be equitable (i.e. prices are dependent on some predetermined, logical and consistent stratification). Therefore, based on the current knowledge and within a liberal context, using epigenetic profiles to stratify the consumers’ premiums might lead to contexts in which discrimination based on epigenetic characteristics is produced. In particular, even if legal provisions created in several states and communitarian institutions prevent discrimination in general (European Parliament 2000) and on the base of genetic characteristics (German Ethics Council 2013; Slaughter 2007), that often equate to epigenetic information, this might not translate to concrete avoidance of discriminations for persons living in daily social contexts.

We can instead consider whether corporations should take responsibility for the health of individuals. In this scenario, if we assume that our patient’s increased cancer risk is due to his unhealthy behaviours, the tobacco, alcohol and fast food industries are all contributing to his potential to acquire ill health, both through making their products accessible and through their advertising campaigns. Would it be reasonable to expect these actors to assist members of the society to implement behavioural change? And if so, what kind of model might this follow? One possibility might be that the taxation of the corporations’ profits producing and trading toxicants, like plastics, dyes, tobacco, oil or carbon, would be allocated to fund those parts of the healthcare system that might take care of those people suffering from diseases caused by those chemicals. Nevertheless, given that transnational companies do not want to be considered as liable for the increase of number of persons living with and dying from diseases caused by those chemicals (Chapman 2004; Hirschhorn 2004), at what level this negotiation should take place is highly problematic. Indeed, traditional institutional decision-making processes are far from considering other forms of negotiation like environmental conflicts among local residents, civil society groups, private industry profits and public, national and communitarian institutions (Greyl et al. 2013; Martinez-Alier et al. 2016; Perez et al. 2015).

## Scenario 3—prenatal care

A 23-year-old woman, who lives in a low socioeconomic area, goes to a general practitioner because she suspects that she is pregnant. The doctor confirms the pregnancy, the woman’s first, and they discuss her options. She decides to continue the pregnancy, and the doctor discusses lifestyle changes she should make in order to increase the health of the fetus, such as ceasing smoking, avoiding foods considered ‘risky’ during pregnancy (unpasteurised cheeses, uncooked fish, etc.) and also the importance of adequate nutrition. The doctor asks about the woman’s eating habits and identifies that she has a poor diet that is both inadequate in nutrition, to ensure the health of the baby, and is also comprised predominantly of packaged foods. The doctor mentions that there is some evidence to suggest that, from an epigenetics perspective, eating large quantities of foods that have been exposed to particular plastics that contain endocrine disrupting chemicals can carry considerable health risks to the fetus, such as abnormal uterine and cervical development (Bondesson et al. 2009; Brotons et al. 1995; Casas et al. 2011). In addition, the doctor informs her that poor maternal nutrition can result in cardiovascular disease when the child reaches adulthood. The doctor suggests that in order to promote the health of her baby, she should drastically reduce her consumption of packaged products and eat more fresh food.

While in scenario 2 we discussed the responsibilities of an individual patient to implement behavioural change in response to epigenetic information about his own health, here we are focusing on the responsibilities of this young, pregnant woman to implement behavioural change in order to enhance the health of her future child. On the surface, this does not seem very different from the expectations we normally place on women during pregnancy. The internet is riddled with information (and misinformation) about what women *should* do during pregnancy in order to ensure the health of their baby. Women are instructed not to drink alcohol, not to smoke, to eat folate-rich foods, to exercise, to be careful about exposure to kitty litter, to avoid undercooked meat and eggs, to avoid unpasteurised cheeses, to avoid too much caffeine, to eat fish, but not too much fish, etc. However, what differs in this scenario, compared to the standard expectations placed on women to adapt their behaviours to promote the wellbeing of their future child, is that some of the advice the doctor is recommending is to promote the health of the future child based on epigenetics.

To explore whether the mother-to-be in this scenario has any responsibility to change her behaviour in response to this information from her doctor, we must first consider the moral status of the fetus and the obligations of mothers to their unborn children more broadly. Authors have suggested that although women are free to choose whether they want to continue a pregnancy, once a pregnant woman has decided to do so, she, and other members of society, then have fiduciary obligations towards the fetus (McCullough and Chervenak 2008). The determination of the point at which these obligations commence is based on the idea that the fetus is not viable (i.e. able to sustain its life independently), so its ability to become a child is dependent on whether the woman decides to continue the pregnancy (McCullough and Chervenak 2008). Although the fetus does not have independent moral status and therefore no ‘rights’, it has dependent moral status, based on the role it is ascribed by the mother-to-be, the doctor and the society. According to McCullough and Chervenak (2008), this dependent moral status means that there are beneficence-based, rather than rights-based, obligations towards the fetus. However, once a mother-to-be has decided to continue the pregnancy, some have postulated that the fetus may then acquire a different moral status—that of a future person—with their own full moral rights (Loi and Nobile 2016). If we accept this argument, then not only would the mother-to-be have a responsibility to act in a way that protects the future health of the fetus but also it would be justifiable for the State to reinforce this if the mother was non-compliant because ‘[…] the interests of future children and adults matter as much as the interests of pregnant mothers’ (Loi and Nobile 2016).

If we accept then that the mother-to-be has an obligation to promote the health of the fetus, we need to consider whether she has the capacity to do so given (a) the reliability of the information she has been provided, and (b) the situation in which she is embedded. In relation to the reliability of the information, there is considerable evidence that exposure of the fetus to high levels of endocrine disrupters leads to disorders in the development of the reproductive system and therefore that exposure to these chemicals should be avoided (Bondesson et al. 2009; Casas et al. 2011; Fernandez et al. 2016; Skinner 2014). There is also evidence to suggest that poor maternal nutrition during pregnancy leads to low birth weight and also increased risks of cardiovascular disease (Barker et al. 1993). Therefore, taking steps to improve her diet is likely to lead to better health outcomes for the child, both in the short and in the long term. But is it realistic to expect her to reduce her intake of packaged foods that contain endocrine disrupting agents and to eat a more nutritious diet? In reality, at the level of the individual and without support, the options for our pregnant woman are quite limited. Firstly, she needs to be provided with information so she can make informed decisions about which foods to choose. Perhaps she has never been educated as to which foods have greater nutritional value or taught how to cook good quality meals, because this is how her parents ate. She might also currently live with the father of the child-to-be who also works long hours and has poor knowledge of what constitutes a good diet, and is therefore not going to be able to provide support in her attempts to change her diet. In addition, the information that she receives from the doctor might be quite confusing for her because, at first glance, to advise someone to both increase food intake and also to restrict intake of *particular* foods might seem contradictory. This could result in further reduction in food intake by not eating packaged foods without replacement with more nutritious foods, increasing the overall risk of cardiovascular defects for the child-to-be.

If we think about the role of the doctor in this scenario, they might feel that they have informed her of the risks to her future child so she is empowered to enact change to improve their health. However, perhaps all they have done is place the burden of responsibility on the woman, making her anxious about a situation that she is not in a financial or social position to change. Or perhaps she will attempt to change her eating habits. Although the doctor may feel that he fulfilled his medical obligations, one might consider the doctor irresponsible to disclose this kind of information without also providing assistance in implementing behavioural change. But what kinds of solutions might actually be beneficial in this situation?

Although her doctor might be able to provide her with some of the educational aspects, she also needs to be able to access and afford the healthy options if she chooses to do so. We know that she has a low socioeconomic status, so it may be difficult for her to afford to buy fresh produce when often packaged and processed foods are much cheaper, due to their poorer quality. Perhaps she works very long hours and eats these sorts of meals because they do not require much cooking time. If she is quite motivated, she might, for example, start driving to a different supermarket, which is an extra 20 minutes away, in order to shop for fresher foods. But this takes more time, so she has less time to cook meals than she already had, which means she misses out on sleep, which is also not healthy for her or the fetus. Or maybe she will start buying fresh foods rather than packaged ones from her local supermarket. But this is more expensive, and she needs to work even longer hours to cover the costs, which has the same effect. All of these factors mean that her ability to implement behavioural change in order to enhance the health of the fetus might be impaired. But does the fact that it might be difficult for the mother-to-be to enact change mean that she should not be informed of her potential to do so? While one might argue that informing her may place an unrealistic burden on her, on the other hand, not passing on this information removes any possibility for her to improve the future health of her child.

A number of authors have drawn attention to the inaccurate weighting, and therefore unfair responsibility, that is placed on the maternal contribution to the disease states of their future children (Hedlund 2012; Kenney and Müller 2016; Richardson et al. 2014). As Richardson et al. state, there is ‘the need for societal changes rather than individual solutions’ (Richardson et al. 2014). Therefore, in order to provide the kind of support this pregnant woman needs, we need to think about the potential for other actors to assist at the societal level. For example, the public health sector has an interest in having a healthier population, both because they have the ultimate goal to foster the right of members of the society to health and also because this places less of a burden on hospitals. Therefore, although education could be provided to an individual woman by a health practitioner, one could also consider whether larger health institutions could organize educational sessions for pregnant women in order to target more of the population, as implemented in Denmark (Lemus 2015). One might consider giving food allowance vouchers to pregnant women to shop in organic food stores. However, if we think about the mismatch model, then this means that once the child is born and the food vouchers cease, children may not be epigenetically ‘programmed’ to their environment. Instead, perhaps representatives could assist pregnant women living in urban areas to establish a farmers’ market by giving them guidance and connecting them with local producers. This would create ongoing access to fresh food by adopting a ‘teach a man to fish’ mentality. Policy-makers could also play a role in assisting pregnant women to avoid eating packaged foods, such as developing policies which place pressure and obligations on food producers to reduce the use of harmful plastics. Of course, the development of these interventions and support systems should always involve discussions with the members of the society who need them in order to understand the problems and ensure that the strategies are appropriate to the population. Therefore, it would be important to set up a dialogue with women who might want to change their diet to determine precisely which barriers are preventing them from achieving this behavioural change. Only then can the State effectively implement these strategies. Nevertheless, in those cases in which a negotiation takes place, citizens, consumers and lay people might not understand, or even be confused, by policy-makers and experts. Indeed, scientific opinions and regulations of chemicals such as endocrine disruptors can be contradictory. Take for instance the 2010 statement produced by the European Food Safety Authority (EFSA) about Bisphenol A, a plastic used in food packaging that is an endocrine disruptor (EFSA Panel on food contact materials and processing 2010). The 2010 statement produced by EFSA declared the safety of Bisphenol A. In contrast, in 2010 the Danish Environmental Protection Agency (EPA), instead prohibited the use of Bisphenol A in all food contact material for children aged 0-3 years. On the base of studies showing the exponential effects on human health of combination of endocrine disruptors, in 2012, the Danish EPA banned four phthalates from all consumer products (phthalates are molecules used, for instance, to soften food packaging, that in combination with Bisphenol A might easily increase health problems of individuals and their children) (Lemus 2015). Or consider the French ban for using Bisphenol A in all food containers, underpinned by the French Agency for Food, Environmental and Occupational Health & Safety (ANSES). In this specific case, there is an explicit conflict between national and communitarian institutions that make scientific opinions and policies less understandable for experts and lay people. There are various solutions here. for instance, local communities might organize meeting days with information materials, scientific experts and institutions based in the same area, to proactively adapt to specific, situated contexts. At the same time, it might be important to consider *dissenting* opinions and facts on biomedical research and public health policies, in order to actively engage citizenry and lay people in science and politics. Last but not the least, it is important that scientists, technicians and researchers embrace a more comprehensive analysis (i.e. compared to the airy and principlistic approach of bioethics) of the issues produced in the science/society interaction.

## Scenario 4—the workplace

The CEO of a power plant has received reports that a number of employees have recently been diagnosed with a range of cancers affecting various different tissues, such as the lungs, skin and bladder, and various oral and esophageal carcinomas. Their medical advisor suggests that this may be due to exposure to benzo[a]pyrene (BaP), a polycyclic aromatic hydrocarbon that is produced through incomplete combustion (Tong et al. 2006) and classified by the International Agency on Research on Cancer (IARC) as carcinogenic in animals and humans (IARC 2010b). BaP is lipid soluble, accumulates in adipose tissue and is transferred across the placenta and the fetal blood-brain barrier (Brown et al. 2007; Hood et al. 2000). BaP has shown both genetic and epigenetic toxicity (Perera and Herbstman 2011). Moreover, BaP is an endocrine disruptor - a steroid-mimicking chemical affecting fetal growth (Choi et al. 2006), cognitive development and behavioural disorders. Studies on animals have shown that BaP interferes with early brain development, peripheral lymphocyte development and causes alterations in levels of noradrenaline, dopamine and serotonin (Konstandi et al. 2007; Stephanou et al. 1998; Tekes et al. 2007). Concerned both for the other employees, and also for the reputation of the company, the CEO, along with the board, decides that all employees must submit their samples for epigenetic testing in order to assess their current DNA methylation levels. Those who are shown to have low global DNA methylation levels will be given a payout but will lose their jobs, because they are considered to be at high risk of cancer and should not continue to be exposed to BaP. Those with more normal levels of DNA methylation will be allowed to remain in their positions. However, they will be required to sign new contracts where they commit to health-promoting behaviours, such as exercise and good diet in order to combat their BaP exposure.

In this case, epigenetic tools can have several non-overlapping potential uses and the interventions developed following epigenetic testing could serve a gamut of solutions, each focusing on a different level. One solution might focus on the worker as not being epigenetically adapted to a specific, toxic environment. Another might focus on the workers’ habits conceived as a means to individually adapt and cope with damaging pollutants. Alternatively, the focus could be on the company as responsible for damaging the environment, its inhabitants and especially the workers. Here, the major issue is the compatibility, or lack thereof, of all these uses of epigenetic testing. Is it possible to, at the same time, protect the health of the workers, the industrial activities and the population more broadly, including the workers’ families?

As we have outlined in previous scenarios, it is unfair to place all of the responsibility on the individual workers for their own health, as the employers here are doing by making them sign a contract committing them to undertake activities to optimize their health in response to the risks posed by their work environment. Because they have greater power, corporations need to take on more responsibility for the health of their workers. But what actions might be possible in response to this scenario? It is possible that both the groups of workers, those who were fired and also those asked to sign a contract, might decide to initiate a lawsuit against the employers. Those who were fired might invoke legal intervention on the base of their right to work. Perhaps the factory is in an economically depressed area that is disbanding most of its industrial sites and without social measures of welfare to guarantee the fired workers either a decent subsidy or alternative jobs. Those who were obliged to sign a new contract might decide to file a lawsuit against the employers on the basis of violation of the environmental law on health and safety of workers. In this case, those most motivated to initiate the lawsuit might be the workers’ family members, specifically their partners and children, because the workers themselves are under occupational blackmail, being forced to choose whether to live or to work. We need to also consider whether epigenetic testing ordered from the employers is discrimination operated at the workers’ expense. Should the company have the right to ask employees their health status in order to fire those who might already have been damaged from the pollutants? Can we invoke laws and regulations, such as the ‘Genetic Information Nondiscrimination Act’ that prohibits employers from asking and using the individuals’ genetic information when making hiring, firing or job placement (Slaughter 2007)?

We could envisage this legal action as a negotiation between all the actors interested in the rights and welfare of workers and citizens, which might be lacking in some countries. We can imagine that a regional agency that takes care of the environmental protection, together with some grassroots movements that want to protect the people living in the area surrounding the plant, might also enter the scene. These grassroots movements may initiate a massive media campaign to encourage the public to boycott the company. These actors from the civil society aiming at enhancing public and communal goods might then push institutional bodies to order other tests in order to analyse the association between epigenetic dynamics present in the workers’ samples with exposure to BaP. On the basis of these outcomes, the regional agency might order the closure of the plant to convert it into a more sustainable and less polluting activity, a move that would also cut many job positions.

A theoretical point is slowly emerging to overwhelmingly disrupt the existing tradition of epidemiology and public health. Historically, epidemiological knowledge that was meant to be generalizable for most animals and human populations, derived from in silico, in vitro, in vivo, logical/mathematical models, cell cultures, model organisms and cohorts of humans, has been translated into public policies which are meant to be universally applicable. However, epigenetics, by some of its accounts, seems to say something different and points to the capability of each specific organism to cope with a specific environment. Is the focus of epigenetics on individual biological plasticity challenging those preventive policies developed by communitarian agencies on pollutants of various kinds, habits or jobs (Davis 1986)? Now, the entity causing a disease may not only (and not primarily) be a specific molecule or human behaviour but also the genetic or epigenetic susceptibility of a person to that specific disease, e.g. a specific epigenetic makeup, programmed in the early phases of development, that may eventually not match with a specific environment. Within this ‘mismatch’ aetiological model, what are the responsibilities given to those actors or factors that shaped the two, non-matching environments (i.e. the perinatal environment that programmed the individual, and the environment in relation to which the adult develops the disease)? Is there a resurgence of the importance given to plasticity of an individual’s biological makeup in spite of environmental, sociocultural factors? Will biological plasticity be used to rank individuals, classes, genders, etc. as was proposed some decades ago, by right-wing Lamarckians (Meloni 2016b)?

## Discussion

In all these four scenarios, we have shown that epigenetic testing is mainly used to scrutinize the relationship between individuals, public and private institutions, future generations and the environment, be that material or social in nature. Depending on the context in which epigenetic testing is embedded, these relationships will carry with them certain roles, and therefore responsibilities, for the actors involved. Compared to the sociological notion of genetic responsibility, where the emphasis is on individuals, as we have illustrated, epigenetic responsibility could instead redistribute roles within the community. In this sense, epigenetics may allow for better realization of the relational concept of responsibility. Indeed, the biological concept of inheritance has been reshaped by epigenetic studies (Gilbert 2011; Gilbert and Epel 2009; Meloni 2016a). During the last century, we have witnessed the birth of, and increasing importance given, to genetics and individual agency. This normative genetic shift corresponds with changes in the moral obligations of individuals, withdrawal of solidarity and reduction of professional responsibility (Schicktanz 2016). If epigenetics is used within the same ideological framework where the agency of individuals plays a main role to the detriment of collective agency, then other important concepts will be reshaped and responsibilities reallocated. What we have tried to sketch here is the use of epigenetic tools and models within dialogical scenarios where different actors from several levels of the society are considered. We have focused mainly on the agency of individuals, corporations and the State; concepts that are often overlooked within the current scientific literature and discourse on epigenetics.

Caring for oneself, for future generations and for environmental protection, are aspects which are interlinked and pertain to interactions among individuals, the State and the private sector, and are under negotiation at a global scale. These three notions, and their interactions, challenge the individuals’, communities’ and public or private entities’ conception of time relating to the *length and effects of an event* (e.g. what effect does the quantity and quality of diet have for a person’s health for the next month, versus for the next 20 years of health of her child?). The interaction between the three notions of individuality, next generations and environment also raises questions regarding who should be the *moral agent* to whom responsibilities are allocated. For example, are workers or citizens responsible for their own health, or should the employers, the industry and the State also be considered as responsible for certain environments that contribute to diseases? In addition, the interweaving of these three notions to redistribute responsibilities is captured by the *temporal direction* (backwards or forwards) considered by the scientific enquiry, such as whether researchers should focus on preventive policies to help people not get sick or should they instead focus on developing therapeutics to cure and care for persons with diseases? And how should limited research resources be allocated between these two views? Allocation of responsibilities is a process following *norms* that are under the supervision of authorities that are defined within specific forms of government and at the State, supranational or corporate levels. Moreover, the norms used to allocate these responsibilities might be used to produce *regulations* in which processes, actors or subjects will be considered, such as whether emphasis could be placed either on scientific/epistemic norms or on social norms. For example, should scientific practices and theories impacting directly, and at different levels on the people’s lives, be discussed through norms developed by the civil society or are scientific/epistemic norms sufficient to regulate science and its effects on society?

In some of the scenarios in this paper, we have situated our point of view sympathetically with certain scientific ‘truths’, such as that hard drinking and smoking is an unhealthy habit for men and women, whether they are pregnant or not. This might make it difficult for the reader to disentangle epistemic truths from philosophical, ethical and moral arguments. This of course might be considered either a limitation or a point of advantage, depending on their point of view. On one hand, having plausible case scenarios and scientifically informed stories might improve the comprehension of practices and ideas. On the other hand, being partisan on specific scientific truths might propose a simplistic picture of science, in which facts are instead both realistic and constructed, depending on negotiations and interests of stakeholders. As an example, a molecule like Bisphenol A is, to date, considered toxic by some countries like France or Denmark but not by the European Union to whom these two countries belong. At the same time, considering a scientific fact as true might obscure the moral, ethical and political aspects of concrete situations, reducing these latter aspects to epistemic arguments, and leading to obligations and ethical imperatives. Furthermore, as epigenetics was developed in a specific period of time in which the States were less challenged by translational corporations and globalization, it is of primary importance to consider the models and practices of epigenetics within specific contexts where international networks of research can be aligned to interests of different actors, such as national or supranational public institutions, translational corporations or foundations and grassroots movement of citizens.

We have challenged the importance given to individual agency, both in practice and as a concept, in that it does not allow for concrete possibilities of action for individuals. Indeed, being included in a framework of liberal governance, epigenetics is mainly used to discipline individuals considered as isolated from their social and economical contexts (Santoro 2010). Here, we instead propose a model in which a dialogical relationship among collective, individual, private and public agencies, is put in motion. As we have shown, epigenetics can be used to foster either individual or social rights. Trying to establish an equilibrium between social and individual rights by means of epigenetics practices might be a manner to foster social justice.
